# Novel Immunotherapeutics for the Treatment of Non-Small Cell Lung Cancer (NSCLC) Resistant to PD-1/PD-L1 Inhibitors

**DOI:** 10.3390/cancers16213603

**Published:** 2024-10-25

**Authors:** Jyoti Malhotra, Amy Huang, Arya Amini, Percy Lee

**Affiliations:** 1 Department of Medical Oncology & Therapeutics Research, City of Hope National Medical Center, Duarte, CA 91010, USA; 2 Department of Medicine, University of Connecticut School of Medicine, Farmington, CT 06030, USA; amhuang@uchc.edu; 3 Department of Radiation Oncology, City of Hope National Medical Center, Duarte, CA 91010, USA; aamini@coh.org (A.A.); percylee@coh.org (P.L.)

**Keywords:** trials, immunotherapy, resistance

## Abstract

Immunotherapy with PD-1/PD-L1 inhibitors is the standard method of care for the treatment of newly diagnosed advanced or metastatic NSCLC, with or without chemotherapy. Many tumors, however, develop resistance to these immunotherapy agents. There is a need to develop more effective therapies for patients with metastatic NSCLC in the second-line setting and beyond. In this review, we present an overview of novel immunotherapies being investigated regarding the treatment of these patients. We summarize completed, as well as ongoing, trials investigating these therapies as monotherapy or in combination with PD-1/PD-L1 inhibitors. These include immune co-stimulatory antibodies, T-cell agonists, oncolytic viruses, vaccines, TIL therapies, and CAR-T therapies.

## Simple Summary

The standard therapy for advanced lung cancer is immunotherapy with or without chemotherapy. In this review, we summarize clinical trials that have been completed or are ongoing that concern the treatment of advanced lung cancer which has been treated previously with standard immunotherapies and has become resistant to these. Many different therapies such as vaccines, immune checkpoint therapies, and cellular therapies are currently in development, but the majority are still in early-phase trials.

## 1. Introduction

**Figure 1:**
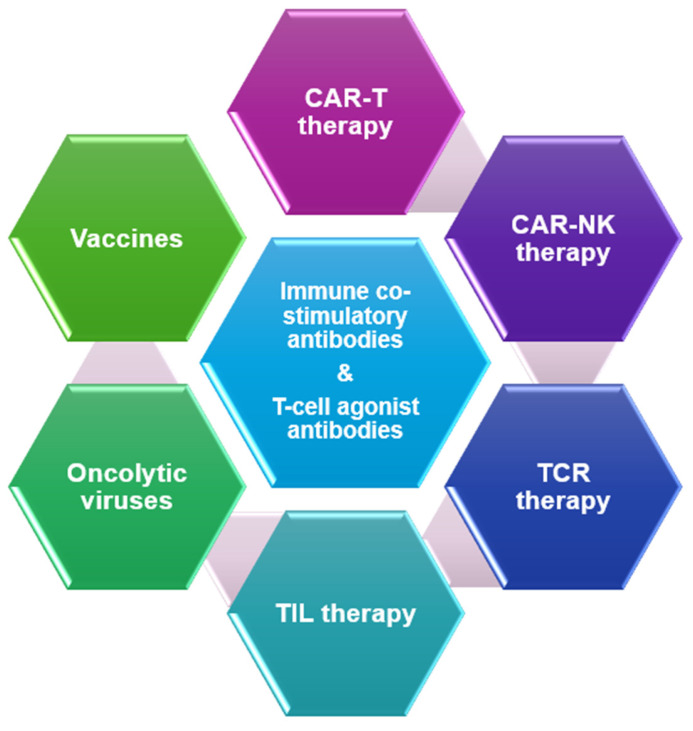
Therapeutic approaches for PD1/PD-L1-resistant NSCLC.

Immunotherapy with Programmed Cell Death Protein/Programmed Cell Death Ligand 1 (PD-1/PD-L1) targeting antibodies has significantly improved clinical outcomes for patients with non-small cell lung cancer (NSCLC) and has become an integral part of standard treatment regimens. The response to PD-1/PD-L1 inhibitors varies, and many tumors develop resistance to immunotherapy. Resistance to immunotherapy can present itself during initial treatment (primary resistance) or may develop after some clinical benefit (secondary or acquired resistance) [1]. The tumor microenvironment, particularly the presence and activity of T cells, also significantly influences resistance to immunotherapy, with both tumor-extrinsic as well as tumor-intrinsic mechanisms playing a role [2,3,4,5]. In this review, we discuss novel and emerging immunotherapies and combinations that are being investigated regarding the treatment of Pd-1/PD-L1-resistant advanced NSCLC (Figure 1).

## 2. Immune Co-Stimulatory Antibodies

The T-cell-mediated immune response against cancer cells is initiated through antigen recognition by the T cell receptor (TCR), which is controlled by a balance between co-inhibitory and stimulatory signals [6,7]. Although the PD-1/PD-L1 and Cytotoxic T-lymphocyte associated protein 4 (CTLA4) pathways are the two most studied immune checkpoints, many of the other immune regulatory checkpoints are currently being investigated. More recently, bispecific antibodies that simultaneously target PD-L1 and other immune regulatory molecules are also being investigated in patients with PD-1-resistant NSCLC [8]. One potential challenge when developing bispecific antibodies is that their efficacy may be limited if there is insufficient T-cell infiltration in immune cold tumors [8]. Cadonilimab is a bispecific IgG-single-chain Fv fragment antibody that binds to PD-1 and CTLA-4 [9]. In a multicenter phase Ib/II trial, patients with pre-treated NSCLC were enrolled in three cohorts: immunotherapy naïve; patients with primary resistance to immunotherapy; and patients with acquired resistance to immunotherapy [9]. The objective response rate (ORR) was 10% in the PD-1-naïve cohort, but no responses were observed in the PD-1-resistant cohorts. Thus, cadonilimab demonstrated limited efficacy in patients with resistance to immunotherapy.

T cell immunoglobulin and ITIM domains (TIGIT) is a co-inhibitory receptor expressed on immune cells such as NK cells, effector T cells, and regulatory T cells (Tregs) [10]. TIGIT is also co-expressed with PD-1 on exhausted T cells and may provide a strategy to restore T-cell immunity [11]. Several anti-TIGIT antibodies are currently in development for PD-1-resistant NSCLC. A phase I trial with an anti-TIGIT antibody, vibostolimab, reported an ORR of 26% when combined with pembrolizumab in PD1-naive patients with NSCLC, but there was minimal efficacy in the PD-1-resistant cohort (ORR 3%) [12]. AZD2936 is a bispecific, humanized antibody targeting PD-1 and TIGIT. In an interim analysis of 80 patients enrolled in the ARTEMIDE-01 trial, AZD2936 showed an acceptable safety profile in patients with PD-1-resistant advanced NSCLC [13]. Among 76 evaluable patients, 3 patients had a partial response and 30 patients had stable disease. Further data are awaited (NCT04995523). HB0036 is a bispecific IgG1 antibody targeting both PD-L1 and TIGIT. In a phase I/II trial of HB0036 (NCT05417321) in patients with PD-1-resistant advanced solid tumors, no dose-limiting toxicity (DLTs) was observed. The disease control rate was 69%, with a durable response lasting more than 36 weeks in a patient with lung sarcomatoid carcinoma [14]. A phase I/II study of HLX301, a recombinant humanized anti-PDL1 and anti-TIGIT bispecific antibody, is currently enrolling patients with advanced solid tumors, including NSCLC (NCT05390528).

Lymphocyte activation gene 3 (LAG3) is an inhibitory receptor expressed on activated T and NK cells that inhibits the function of CD8+ effector T cells, as well as the function of Tregs [15]. The phase II CITYSCAPE study evaluated the use of the anti-LAG3 antibody, tiragolumab in combination with anti-PDL1 antibody, atezolizumab, as compared to the use of a placebo plus atezolizumab, as a first-line treatment for patients with PD-L1-positive advanced NSCLC. A higher efficacy was reported with the combination compared to atezolizumab alone (ORR 31% versus 16%, and median PFS 5.4 months versus 3.6 months; *p* = 0.015) [16]. Based on this, the phase III SKYSCRAPER-01 study was initiated for patients with PD-L1-high advanced NSCLC who did not meet the co-primary PFS end point (NCT04294810). Eftilagimod alpha, a soluble LAG-3 protein, acts as an MHC class II agonist triggering the activation of antigen-presenting cells (APC) and CD8 T-cells. In the TACTI-022 trial involving 26 patients with PD-1-resistant disease, ORR and DCR were 8.3% and 33%, respectively, with eftilagimod alpha [17]. Most patients (~83%) showed either deceleration in tumor growth or shrinkage of target lesions. HLX26 is a novel humanized anti-LAG3 monoclonal antibody [18]. In a phase I trial, patients with refractory solid tumors including NSCLC were treated with escalating doses of HLX26 plus anti-PD1 serplulimab every 3 weeks [18]. Of the nine treated patients (four with NSCLC), three patients had the best overall response (BOR) of stable disease. A phase I dose-finding study of RO7247669, an anti-PD-1 and anti-LAG-3 bispecific antibody, is also currently enrolling participants (NCT04140500).

TIM-3 is another inhibitory molecule like CTLA-4 and PD-1. The AMBER trial (NCT02817633) is evaluating cobolimab both as monotherapy and in combination with PD-1 inhibitors in advanced solid tumors [19]. COSTAR Lung (NCT04655976) is another trial involving patients with PD-1-resistant NSCLC that is investigating cobolimab plus anti-PD1, dostarlimab, and standard of care chemotherapy (docetaxel). AZD7789, an anti-PD-1 and anti-TIM-3 bispecific antibody, is being investigated in a phase I/IIA trial involving patients with advanced solid tumors (NCT04931654).

## 3. T-Cell Agonist Antibodies

Agonist antibodies against co-stimulatory molecules such as 4-1BB (CD137), OX40 (CD134), and ICOS (CD278) are being investigated in combination with anti-PD-1 immunotherapy, but clinical responses have been modest to date [11]. The inducible co-stimulator (ICOS) of T cells is upregulated following initial T-cell priming and induces a signal through the PI3K/protein kinase B pathway, resulting in T-cell proliferation and survival [20]. The phase I/II ICONIC trial evaluated the ICOS agonist vopratelimab, alone and in combination with nivolumab, in patients with advanced solid tumors. The use of vopratelimab resulted in a poor ORR of 1.4% when used alone and 2.3% in combination with nivolumab [21]. The prospective selection of ICOS-positive tumors did not enrich the responses. A phase II trial was initiated that included NSCLC patients (SELECT, NCT04549025), but eventually the drug program was discontinued.

OX40 is an immune costimulatory receptor expressed on activated CD4+ and CD8+ T cells that promotes T cell proliferation and survival in the tumor microenvironment [22]. BGB-A445 is an agonist antibody that does not compete with endogenous OX40 ligand binding. In the dose escalation part of a phase I trial (NCT04215978) of BGB-A445 alone or in combination with anti-PD1 tislelizumab, for patients with advanced solid tumors, the ORR was 23% [22]. Further results from the trial are awaited. INBRX-106 is an agonistic, anti-OX40 antibody being investigated in combination with pembrolizumab in a phase I/II trial (NCT04198766). Multiple cohorts, including PD-1-resistant NSCLC patients, are being enrolled. 4-1BB is a costimulatory receptor that is upregulated on tumor-infiltrating lymphocytes (TILs), which promote T-cell proliferation and activation [23]. INBRX-105 is a 4-1BB and PD-L1 bispecific antibody, and the cross-linking of PD-L1 to 4-1BB by INBRX-105 leads to conditional 4-1BB activation at sites of high PD-L1 expression, potentially limiting toxicity [23]. NCT03809624 is a phase I trial with multiple cohorts including in PD-1-resistant NSCLC patients, but it is no longer enrolling participants. GEN1046 is a PD-L1 and 4-1BB bispecific immunotherapy designed to act on both pathways by combining simultaneous and complementary PD-L1 blockade with conditional 4-1BB stimulation in one molecule [24]. A phase I trial using GEN1046 as monotherapy as well as in combination with pembrolizumab in patients with PD-1-resistant NSCLC is ongoing (NCT05117242).

## 4. Vaccine Therapy

Antigenic target vaccines comprise immunogenic tumor antigens or cells administered together with an immunoadjuvant to enhance anti-tumor immune response [25]. Tumor antigens may be tumor-associated antigens (TAA) or neoantigens arising from mutations in tumor DNA [25,26]. NEO-PV-01 is a personalized cancer vaccine consisting of up to 20 synthesized peptides of 14–35 amino acids derived from the patient’s mutated tumor DNA [27]. The NEO–PV–01 vaccine is administered subcutaneously, via injection, after mixing with adjuvant poly-ICLC. In a phase I trial that used NEO-PV-01 in combination with pemetrexed, carboplatin, and pembrolizumab as a first-line therapy for advanced non-squamous NSCLC, 38 patients were treated with the regimen [27]. De novo neoantigen-specific CD4+ and CD8+ T cell responses were observed post-vaccination, and the regimen was tolerable. OSE2101 is a T-cell epitope-based cancer vaccine designed to induce cytotoxic T cells against five TAAs frequently overexpressed in NSCLC (HER-2/neu, CEA, MAGE 2, MAGE 3, and p53) [28]. TALANTE-1 was a two-step trial involving patients with HLA-A2-positive PD-1-resistant advanced NSCLC (*n* = 219), with treatment randomized between OSE2101 and chemotherapy (docetaxel or pemetrexed). In April 2020, a decision was taken to prematurely stop the accrual due to COVID-19. In the interim analysis, the median OS favored OSE2101 over chemotherapy, but was not statistically significant (*p* = 0.36) [28]. In the acquired resistance subgroup, OSE2101 significantly improved the median OS versus chemotherapy (11.1 vs. 7.5 months; *p* = 0.017). A phase III trial randomly assigning patients to OSE2101 or chemotherapy cohorts is now planned for patients with HLA-A2-positive metastatic NSCC and acquired resistance to immunotherapy (ARTEMIA; NCT06472245).

CIMAvax-EGF is a vaccine comprising a chemical conjugate between EGF and P64, a recombinant protein formed from *Neisseria meningitides* and an adjuvant [29]. Patients with PD1-naïve, pre-treated NSCLC were enrolled in a phase II trial and received CIMAvax-EGF every 2 weeks, receiving four doses in combination with nivolumab, followed by monthly maintenance with CIMAvax-EGF and nivolumab every 2 weeks [29]. The disease control rate was 47.6% with a median OS of 11.9 months. CIMAvax-EGF is currently being investigated in combination with pembrolizumab as maintenance therapy after first-line chemoimmunotherapy for NSCLC (NCT02955290). BNT116 is an intravenously administered RNA-lipoplex cancer vaccine comprising six RNAs, each encoding a TAA frequently expressed in NSCLC. LuCa-MERIT-1 is a phase I trial using BNT116 alone or as combination therapy with cemiplimab, docetaxel, and/or carboplatin plus paclitaxel [30]. Preliminary results from the first 18 patients (*n* = 13 monotherapy; *n* = 5 cemiplimab added after Cycle 3) treated during the trial reported a tolerable safety profile. Adverse events (AEs) include pyrexia (67%), chills (50%), and vomiting (28%). Six of ten evaluable patients had stable disease. Further enrolment in this trial is currently ongoing (NCT05142189). A phase I trial is exploring the combination of cemiplimab with BNT116 as compared to cemiplimab alone for the first-line treatment of advanced NSCLC and PD-L1 ≥ 50% (NCT05557591). Another single-institution phase I study is investigating the use of a pooled mutant-KRAS peptide vaccine with poly-ICLC adjuvant in combination with nivolumab and ipilimumab in the first-line treatment of advanced NSCLC (NCT05254184).

## 5. Oncolytic Viruses (OV)

OVs are naturally occurring or genetically modified viruses that have been engineered to selectively cause tumor cell lysis while sparing normal host cells using several strategies [31]. The use of attenuated vectors or less virulent strains of viruses may prevent acute or chronic infection [31]. OVs have demonstrated a tolerable safety profile in completed trials so far and have also demonstrated the ability to modify the tumor microenvironment and lyse tumor cells [31,32]. In NSCLC, clinical development of OVs has been limited by a lack of significant benefit from OV monotherapy and by difficulty in achieving adequate viral load at tumor sites. One strategy to potentially overcome this involves exploring novel combinations with OVs, especially with checkpoint inhibitors, and leveraging novel viral delivery systems [32]. Coxsackievirus A21 (Cavatak; CVA21), a naturally occurring human picornavirus, causes mild cold-like symptoms in humans. In the phase Ib STORM trial (NCT02043665; KEYNOTE-200), patients with advanced solid tumors were treated with CVA21 in escalating doses as either monotherapy or in combination with pembrolizumab [33]. There were no dose-limiting toxicities (DLTs), and all patients had detectable anti-CVA21 neutralizing antibodies by day 22 of the study. The ORR was 9% in the NSCLC expansion cohort (*n* = 43) [34]. Although the virus was detected in tumor tissues and there was some increase in PDL1 expression on paired tumor biopsies, the efficacy was not greater than that observed in previous studies with pembrolizumab monotherapy. CV301, a poxviral-based vaccine, has been evaluated in a phase I clinical trial and was shown to be safe and immunologically active [35]. Patients with advanced non-squamous NSCLC received two priming doses of modified vaccinia Ankara-BN-CV301, followed by boosting doses of fowlpox-CV301 for up to 17 doses in combination with nivolumab or pembrolizumab [35]. Of eleven evaluable patients, one patient (9%) had a complete response, one patient (9%) had a partial response, and nine patients (82%) had stable disease. VSV-IFNβ-NIS is a vesicular stomatitis virus (VSV)-based OV that is being tested in combination with pembrolizumab in patients with refractory NSCLC or neuroendocrine cancers (NCT03647163).

Adenovirus is a non-enveloped, double-stranded DNA virus. It has a large linear genome that allows the incorporation of long DNA sequences, thus permitting multiple engineered modifications [31]. A phase II study investigated intratumoral injection of the oncolytic virus ADV/HSV-tk (adenovirus-mediated expression of herpes simplex virus thymidine kinase) followed by stereotactic body radiation therapy (SBRT) at the same tumor site in patients with both PD-1-naïve and PD-1-resistant stage IV NSCLC [36]. The ORR was 29% and the clinical benefit rate (CBR) was 62% in the PD-1-naïve group.

In the PD-1-resistant group, the ORR was 14% and the CBR was 64%. Gene-mediated cytotoxic immunotherapy (GMCI) is another approach in which an adenovirus-based vector expressing the thymidine kinase gene (aglatimagene besadenovec, AdV-tk) is locally administered followed by the anti-viral drug valacyclovir. A phase I dose escalation trial investigated GMCI in combination with intrapleural ADC-tk followed by chemotherapy in 19 patients with malignant pleural effusion from metastatic solid tumors (mesothelioma, NSCLC, and breast cancer). There were no DLTs reported. Of the four patients with NSCLC, three patients experienced durable stabilization of disease, with one patient continuing with follow-up for 29 months after therapy [37]. A phase II study of GMCI in combination with anti-PD1 immunotherapy for patients with PD-1-resistant NSCLC is currently enrolling participants (NCT04495153). MEM-288 is a conditionally replicative oncolytic adenovirus expressing human IFNβ and a recombinant membrane-stable form of CD40L [38]. In a phase I trial involving patients with refractory solid tumors, including NSCLC (*n* = 11) [38], MEM-288 was administered intratumorally once every 3 weeks. Of the 10 evaluable patients, 4 experienced shrinkage of the injected tumor. Several patients also underwent the stabilization or shrinkage of distal non-injected lesions. Biopsies showed decreased tumor cells, increases in CD8+ T cells, increases in T cell clonal diversity, and increases in TCF1+ stem-like CD8+ T cells. An expansion of this trial is currently enrolling patients with PD-L1-resistant NSCLC to test the combination of MEM-288 and anti-PD1 immunotherapy (NCT05076760).

## 6. Tumor-Infiltrating Lymphocytes (TILs)

For TIL therapy, tumor-specific T cells are isolated via resection, activated in cytokines to restore their functionality, multiplied, and then infused back into the patient [39]. Before reintroducing the expanded TILs back into the patient, lymphodepleting chemotherapy is administered as well as IL2 to promote cell growth [39]. The main barriers of this approach are the need for a significant amount of fresh tissue, the several-week window before patients can start treatment, and the potential loss of tumor specificity in vitro [40]. In a phase I trial involving TILs administered with nivolumab in 20 patients with PD-1-resistant advanced NSCLC, three of the thirteen evaluable patients had a confirmed response [41]. The trial reported complete responses in two patients and the responses were ongoing 1.5 years later. In a phase II multicenter study, lifileucel (LN-145), an autologous TIL therapy, was investigated in patients with pre-treated metastatic NSCLC [42]. Lifileucel was manufactured using tumor tissue from different sites, but lung tissue was predominantly used. The ORR was 21.4% (6 of 28 patients). Responses occurred in tumors with profiles often resistant to immunotherapy, such as those with PD-L1 negativity, low tumor mutational burden, or the presence of the STK11 mutation. Bone marrow suppression, hypotension, hypoxia, and fatigue were the most common AEs. These trials demonstrate that TILs can be a promising therapy for PD-1-resistant NSCLC. Several trials are currently investigating TIL therapy for refractory NSCLC (NCT02133196, NCT05681780, NCT05576077, NCT06060613).

Several novel approaches are also being utilized to improve the safety and efficacy of TILs. ATL001, an autologous clonal neoantigen-reactive T cell (cNET) therapy, is designed to target multiple clonal neoantigens expressed on tumor cells and absent from healthy tissue on a personalized basis, in contrast to gene-modified approaches which are limited to single shared antigens that are not expressed on all cancer cells [43]. The product contains a mixed population of CD4+ and CD8+ T cells, both of which are important for the maintenance of long-term cytotoxic responses [43]. CHIRON is a phase I/IIa study aiming to evaluate the safety and clinical activity of ATL001 in patients with advanced NSCLC (NCT04032847).

Another strategy to improve TIL therapies is the manipulation of the PD-1/PD-L1 axis to potentially enhance efficacy, as well as eliminate immunotherapy’s adverse events. Common gene editing techniques being used include the use of CRISPR-Cas9 and transcription activator-like effector nucleases (TALENs) [44]. A phase I/II trial involving TALEN-mediated PD-1–inactivated TILs (IOV-4001) is currently ongoing, with a plan to enroll 53 patients with metastatic melanoma and NSCLC (NCT05361174). LYL845 is an autologous TIL therapy produced with epigenetic reprogramming, which generates populations of tumor-reactive T cells with stem-like qualities and a more favorable phenotype (including CD8 skewing) [44]. A phase I trial investigating LYL845 in patients with advanced NSCLC, melanoma, and colorectal cancer is ongoing (NCT05573035).

## 7. T-Cell Receptor (TCR) Therapy

TCR-based adoptive therapy utilizes genetically modified T cells that are directed against specific tumor markers [39,45]. TCR therapy usually utilizes TCRs restricted to common HLA alleles, such as HLA-A*02:01 [45]. Tumors targeted by TCR therapy need to express the targeted antigen as well as the corresponding antigen-restricting HLA allele. The first recombinant TCR therapy to be approved was afamitresgene autoleucel targeting MAGE-A4 for HLA-A*02 and MAGE-A4-expressing advanced synovial sarcoma in August 2024 [46]. A phase I trial investigated a novel affinity-enhanced NY-ESO-1-specific TCR in nine patients with NY-ESO-1-expressing solid tumors R [47]. Of the six patients who received a dose of 5 × 10^9^ cells, three patients demonstrated tumor responses. Three patients developed cytokine release syndrome (CRS) and one patient developed grade 3 lung injury. IMA203, an autologous TCR-engineered therapy, uses a novel, pairing-enhanced TCR with high affinity and specificity for the HLA-A*02:01-presented peptide related to PRAME, a potential target for multiple solid tumors including NSCLC. An ongoing trial is currently investigating this agent (ACTengine; NCT03686124). Preliminary results from 38 patients report that AEs were manageable, with the most common events being the expected cytopenias (100%), CRS (92%, 3% grade 3), and ICANS (13%) [48]. In addition, 11 of 18 (61%) patients showed an initial objective response at week 6 post infusion. Further enrolment is ongoing, including cohorts combining TCR therapy with nivolumab. AFNT-211 is a TCR therapy consisting of autologous CD4+ and CD8+ T cells engineered to express HLA-A*11:01-restricted KRAS *G12V*-specific transgenic TCR, the wildtype CD8α/β coreceptor, and a FAS-41BB switch receptor [49]. A phase I/II trial with AFNT-211 is currently enrolling patients with KRAS *G12V*-mutated solid tumors, including NSCLC (NCT06105021) [49]. Similar TCR therapies are in progress for KRAS *G12V*-mutated colon cancer and NSCLC (NCT06043713), TP53 *R175H*-mutated solid tumors (NCT05877599), and KRAS *G12D*-mutated tumors (NCT06218914).

## 8. Chimeric Antigen Receptor (CAR) T-Cell Therapy

CAR T-cell therapy, the adoptive transfer of engineered, CAR-expressing T lymphocytes, was first approved in 2017 for the treatment of resistant lymphoma and acute lymphoblastic leukemia [50]. Since then, multiple CAR T therapies have been developed and are currently being investigated for the treatment of various malignancies. CAR T-cells are composed of three elements: an extracellular antigen-binding domain, an intracellular signaling domain that activates T cells, and a hinge that joins these two [51]. For NSCLC, CAR T-cell trials leverage targets such as mucin 1 (MUC 1), epidermal growth factor receptor (EGFR), ROR1, and mesothelin [51]. A phase I trial has been completed involving the use of anti-MUC1 CAR-T cells with PD-1 knockout through CRISPR-Cas9 in 20 patients with advanced NSCLC [52]. No grade 3–5 AEs or CRS were observed. In total, 11 of the 20 patients demonstrated stable disease as the best response to therapy. An ongoing trial is investigating MUC1-targeting CAR-T cells in solid tumors, including NSCLC (NCT05239143). LYL797 is a ROR1-targeted CAR T-cell therapy currently being investigated in patients with ROR1 who have pre-treated NSCLC and triple-negative breast cancer (NCT05274451). A first-in-human phase I study of autologous, mesothelin-targeted CAR T-cell therapy enrolled patients with metastatic lung cancer with pleural metastases as well as malignant pleural mesothelioma [53]. Intrapleural administration of CAR T cells was safe and well tolerated. Stable disease was sustained for ≥6 months in eight patients; two exhibited a complete metabolic response on PET scans.

Another potential target is epidermal growth factor receptor (EGFR), and a phase I clinical trial investigated EGFR-targeting CAR-T cells generated by the piggyBac transposon system in advanced pre-treated NSCLC [54]. The piggyBac transposon system is a simpler and alternative way to introduce CAR transgenes into T cells. Treatment was well tolerated, with the most common AE being fever. After treatment, eight of nine patients with EGFR-mutated NSCLC showed detectable EGFR-CAR T cells in their peripheral blood. One patient had a partial response to treatment lasting for more than 13 months, while six patients had stable disease. The median OS was 15.6 months. Another phase I trial is investigating EGFR-targeting CAR-T cells modified by chemokine receptor type 5 (CXCR 5) in patients with advanced NSCLC (NCT05060796). The challenges in the development of CAR-T therapy for NSCLC are poor infiltration of T cells in the tumor microenvironment, T cell exhaustion, and the heterogeneity of tumor antigens’ expression [51]. The production of CAR-T cells is expensive and requires a significant amount of time, which puts patients with advanced NSCLC at risk of losing the window of opportunity to start treatment before they experience clinical deterioration [55]. One strategy to overcome some of these challenges is to use a split-CAR design that enables the engineering of multi-input CARs capable of Boolean-logic signal integration [56]. Some clinical trials for these logic-gated CAR T cells are ongoing, such as EVEREST-1, which is evaluating A2B530 in tumors with enhanced CEA expression but loss of HLA-A*A02 expression (colon, pancreatic, and NSCLC tumors; NCT05736731). Another trial (EVEREST-2) is using a similar approach to investigate A2B694, an autologous logic-gated CAR T-cell therapy, in tumors that express MSLN but have lost HLA-A*02 expression, such as colorectal cancer, NSCLC, pancreatic cancer, ovarian cancer, mesothelioma, and other solid tumors that express MSLN and have lost HLA-A*02 expression (NCT06051695).

## 9. CAR NK Cell Therapy

NK cells kill target cells but lack a somatically rearranged and antigen-specific TCR, as seen in CD8+ T cells [57]. The main sources of NK cells include peripheral blood, umbilical cord blood, NK cell lines (such as NK92), and induced pluripotent stem cells [57]. One barrier to CAR-NK cells’ development as a therapeutic strategy is the immune-suppressive effects of inhibitory receptors such as PD1 [58]. CAR-NK cells with a novel chimeric costimulatory converting receptor (CCCR), comprising mainly the extracellular domain of PD1, the transmembrane and cytoplasmic domains of NKG2D, and the cytoplasmic domain of 41BB, have been developed that can switch the negative PD1 signal to an activating signal [58]. These CCCR-modified NK92 cells retain the characteristics of NK cells and exhibit enhanced anti-tumor activity in human lung cancer cell lines. A trial has been conducted in China using this therapy on patients with NSCLC (NCT03656705). A phase I/II trial is currently enrolling patients with MUC1-positive solid tumors, including NSCLC, to investigate CAR-NK cell therapy (NCT02839954). Another trial is investigating the safety and efficacy of anti-Trop2 CAR-NK cell therapy combined with chemotherapy in refractory NSCLC (NCT06454890). NCT05334329 is a first-in-human phase 1 dose-finding study of allogeneic, off-the-shelf, frozen and thawed, tumor-reactive anti-PD-L1 co-stimulated killer cells (TRACK-NK) in patients with PD-1-resistant NSCLC [59]. TRACK-NK cells are PD-L1+ NK cells derived from cord blood and engineered to express soluble IL-15 (sIL-15), with an ability to express high levels of tumor-reactive receptors that recognize “tumor stress”, including DNAM-1, NKp30, and NKG2D. Other potential targets for CAR-NK cell therapy in NSCLC include B7-H3 and cMET, and preclinical studies have shown the potential of these therapies [60,61].

In conclusion, advances in the understanding of tumor biology, as well as the development of sophisticated engineering techniques, have led to the development of several novel therapies targeting immunotherapy resistance in NSCLC, such as personalized cellular therapy for lung cancer patients [44]. Further elucidation of the mechanisms underlying immunotherapy resistance is key to developing future treatment strategies. Several potential methods of targeting these mechanisms of immunotherapy resistance have been tested, but clinically significant benefit is yet to be seen. Many trials are still in the early phase of investigation and several late-phase trials have not met the primary endpoint due to multiple factors, such as heterogeneity in the mechanisms of immunotherapy resistance and a lack of predictive biomarkers to select patients. Ongoing trials which are currently enrolling are listed in Table 1. Validation and integration of genomic and other biomarkers for future drug development is essential for the effective targeting of immunotherapy resistance for the treatment of refractory NSCLC.

## 10. Conclusions

Multiple trials are investigating novel immunotherapy agents, both as monotherapy and in combination, for patients with PD-1/PD-L1-resistant NSCLC. While several trials have reported signs of clinical benefit with these agents, these are early-phase trials which will require validation through findings from larger phase II or III trials. So far, late-phase trials have not yielded clinically significant results, likely due to the heterogeneity in immunotherapy resistance mechanisms. A better understanding of the underlying mechanisms of immunotherapy resistance, as well as the development of biomarkers, is necessary for further progress and to guide future drug development for this patient population.

## Figures and Tables

**Table 1:** Current trials in progress for PD1/PD-L1-resistant NSCLC.

Therapy Name	Trial Phase	Mechanism of Action	NCT Number
	**Co-stimulatory antibodies**		
AZD2936	I/II	PD-L1 × TIGIT bispecific antibody	NCT04995523 (ARTEMIDE)
HB0036	I/II	PD-L1 × TIGIT bispecific antibody	NCT05417321
HLX301	I/II	PD-L1 × TIGIT bispecific antibody	NCT05390528
RO7247669	I/II	PD-L1 × LAG3 bispecific antibody	NCT04140500
Cobolimab	I	Anti-TIM3 antibody	NCT02817633 (AMBER)
Cobolimab	II/III	Anti-TIM3 antibody	NCT04655976 (COSTAR Lung)
AZD7789	I/II	PD-L1 × TIM3 bispecific antibody	NCT04931654
	**T-cell agonists**		
BGB-A445	II	OX40 agonist mAB	NCT04215978
INBRX-106	I/II	OX40 agonist mAB	NCT04198766
ES102	I	OX40 agonist mAB	NCT04991506
GEN1046	II	PD-L1 × 4-1BB bispecific antibody	NCT05117242
	**Vaccine therapy**		
OSE2101	III	Vaccine targeting 5 TAA overexpressed in NSCLC	NCT06472245 (ARTEMIA)
KRAS vaccine	I	Pooled mutant-KRAS peptide vaccine	NCT05254184
CIMAvax-EGF	I/II	Chemical conjugate between EGF and P64	NCT02955290
BNT116	I	RNA-lipoplex vaccine with 6 RNAs, each encoding TAA expressed in NSCLC	NCT05142189 (LuCa-MERIT-1)
BNT116	II		NCT05557591
	**Oncolytic viruses**		
Aglatimagene besadenovec (Adv-Tk)	II	Adenovirus-based vector expressing thymidine kinase gene	NCT04495153
MEM-288	I	Conditionally replicative adenovirus vector encoding transgenes for human IFNβ and recombinant chimeric form of CD40-ligand	NCT05076760
VSV-IFNβ-NIS	I/II	Oncolytic VSV expressing IFNβ and NIS	NCT03647163
	**TIL therapy**		
LN-145	II	Autologous TILs	NCT04614103
	I/II	CD-40L-augmented TIL	NCT05681780
TBio-4101	I	Autologous TILs	NCT05576077 (STARLING)
OBX-115	I/II	IL-15 expressing TIL	NCT06060613
ATL001	I/II	Clonal neoantigen-reactive TILs	NCT04032847 (CHIROS)
IOV-4001	I/II	PD-1-inactivated TILs	NCT05361174
LYL845		Epigenetic reprogrammed TILs	NCT05573035
	**TCR therapy**		
IMA203	I	PRAME-specific TCR	NCT03686124 (ACTengine)
AFNT-211	I/II	HLA-A*11:01-restricted KRAS G12V TCR	NCT06105021
FH-A11KRASG12V-TCR	I	KRAS G12V-specific TCR	NCT06043713
NT-112	I	HLA-C*08:02-restricted KRAS G12D TCR	NCT06218914
NT-175	I	HLA-A*02:01-restricted TP53 R175H TCR	NCT05877599
	**CAR-T**		
P-MUC1C-ALLO1	I	Allogenic CAR-T targeting MUC1-expressing tumors	NCT05239143
LY797	I	ROR1-targeting CAR-T	NCT05274451
	I	CXCR5-modified EGFR-targeted CAR-T	NCT05060796
A2B530	I/II	Logic-gated CAR-T (CEA expression, but loss of HLA-A*A02 expression)	NCT05736731 (EVEREST-1)
A2B694	I/II	Logic-gated CAR-T (MSLN expression, but loss of HLA-A*A02 expression)	NCT06051695 (EVEREST-2)
	**CAR-NK**		
	I/II	anti-Trop2 CAR-NK	NCT06454890
	I/II	MUC1-targeting CAR-NK	NCT02839954
COH06	I	TRACK-NK	NCT05334329

mAB, monoclonal antibody; TAA, tumor-associated antigen; VSV, vesicular stomatitis virus; IFNβ, interferon-beta; NIS, sodium iodide symporter; TRACK-NK, tumor-reactive and anti-PD-L1 co-stimulated killer cells.
